# Hypothermic oxygenated perfusion versus standard treatment of donor hearts in heart transplantation: extended follow-up of the NIHP2019 trial

**DOI:** 10.1093/eurheartj/ehag264

**Published:** 2026-04-16

**Authors:** Filip Rega, Guillaume Lebreton, Marylou Para, Sebastian Michel, René Schramm, Emmanuelle Begot, Katrien Vandendriessche, Christine Kamla, Gino Gerosa, Marius Berman, Udo Boeken, Stephen Clark, Aaron Ranasinghe, Fabio Ius, Alberto Forteza, Aldina Pivodic, Felix Hennig, Sabina Guenther, Pradeep Kaul, Adelheid Goerler, Arezu Aliabadi-Zuckermann, Jan F Gummert, Johan Van Cleemput, Andreas Zuckermann, Christoph Knosalla, Göran Dellgren, Andreas Wallinder

**Affiliations:** Department of Cardiac Surgery, University Hospitals Leuven, Herestraat 49, 3000 Leuven, Belgium; Cardiac Surgery Department, Pitié-Salpétrière Hospital, APHP, Sorbonne University, Paris, France; Department of Cardiovascular Surgery and Transplantation, Bichat Hospital, Université Paris Cité, Paris, France; Clinic of Cardiac Surgery, Ludwig-Maximilians-University of Munich, Munich, Germany; German Center for Cardiovascular, Research, Munich Heart Alliance, Munich, Germany; Clinic for Thoracic and Cardiovascular Surgery, Heart and Diabetes Center North Rhine Westfalia, Ruhr-University Bochum, Bad Oeynhausen, Germany; Cardiac Surgery Department, Pitié-Salpétrière Hospital, APHP, Sorbonne University, Paris, France; Department of Cardiac Surgery, University Hospitals Leuven, Herestraat 49, 3000 Leuven, Belgium; Clinic of Cardiac Surgery, Ludwig-Maximilians-University of Munich, Munich, Germany; German Center for Cardiovascular, Research, Munich Heart Alliance, Munich, Germany; Department of Cardiac, Thoracic, Vascular Sciences and Public Health, University of Padua, Padua, Italy; Department of Cardiothoracic Surgery Royal Papworth Hospital NHS Foundation Trust, Cambridge, UK; Department of Cardiac Surgery, Medical Faculty, Heinrich Heine University, Duesseldorf, Germany; Cardiothoracic Centre, Freeman Hospital, Newcastle upon Tyne, UK; Cardiac Surgery, Queen Elizabeth Hospital, University Hospitals, Birmingham NHS Trust, Birmingham, UK; Department of Cardiothoracic, Transplant and Vascular Surgery, Hannover Medical School, Hannover, Germany; Department of Cardiac Surgery, Puerta de Hierro Majadahonda University Hospital, Madrid, Spain; APNC Sweden, Mölndal, Sweden; Department of Cardiothoracic and Vascular Surgery, Deutsches Herzzentrum der Charité, Berlin, Germany; Department of Cardiothoracic and Vascular Surgery, Charité-Universitätsmedizin Berlin, Freie Universität Berlin and Humboldt-Universität zu Berlin, Germany; Clinic for Thoracic and Cardiovascular Surgery, Heart and Diabetes Center North Rhine Westfalia, Ruhr-University Bochum, Bad Oeynhausen, Germany; Department of Cardiothoracic Surgery Royal Papworth Hospital NHS Foundation Trust, Cambridge, UK; Department of Cardiothoracic, Transplant and Vascular Surgery, Hannover Medical School, Hannover, Germany; Department of Cardiac Surgery, Medical University of Vienna, Vienna, Austria; Clinic for Thoracic and Cardiovascular Surgery, Heart and Diabetes Center North Rhine Westfalia, Ruhr-University Bochum, Bad Oeynhausen, Germany; Department of Cardiology, University Hospitals Leuven, Leuven, Belgium; Department of Cardiac Surgery, Medical University of Vienna, Vienna, Austria; Department of Cardiothoracic and Vascular Surgery, Deutsches Herzzentrum der Charité, Berlin, Germany; Department of Cardiothoracic and Vascular Surgery, Charité-Universitätsmedizin Berlin, Freie Universität Berlin and Humboldt-Universität zu Berlin, Germany; Department of Cardiothoracic Surgery, Sahlgrenska University Hospital, Gothenburg, Sweden; Transplant Institute, Sahlgrenska University Hospital, Gothenburg, Sweden

Ischaemic static cold storage (SCS) remains the standard method for preserving donor hearts before transplantation. Nevertheless, it fails to adequately prevent ischaemic injury adversely affecting post-transplant morbidity and mortality. Hypothermic oxygenated perfusion (HOPE) of the isolated donor organ before transplantation has been introduced as an alternative preservation method.^1,2^ In liver transplantation, comprehensive evidence concludes that HOPE reduces postoperative complications and extends the functional organ lifespan.^3^ The NIHP2019 trial is the first multicentre, randomized controlled study to evaluate the clinical efficacy of HOPE for donor heart preservation. Early results demonstrated a reduction in post-operative complications at 30 days for HOPE compared to static cold storage.^1^ This pre-specified secondary analysis of 12-month follow-up assessed whether the early benefits of HOPE translated into sustained improvements in outcome and post-transplant safety.

The design and methods have been previously described.^1,4^ NIHP2019 (NCT03991923) is an open-label, randomized controlled trial conducted in 15 centres across 8 European countries. After randomization, adult candidates were transplanted with a donor heart preserved with either ischaemic SCS or HOPE using a portable machine perfusion system (XVIVO Perfusion, Gothenburg, Sweden). Transplant procedures and post-operative care were performed in accordance with the standard-of-care practices of the respective centre. The current report focuses on the predefined, key secondary endpoint of the trial consisting of a composite endpoint occurring within 12 months after transplant and including; any cause of death, primary graft dysfunction (PGD) (moderate, severe, and right ventricular),^5^ graft failure, biopsy-proven acute cellular rejection (ACR) > 1R,^6^ and chronic allograft vasculopathy (CAV) ≥ 1.^7^ Additional analyses focused on the individual components of the composite measure.

The statistical analysis plan was finalized prior to the database lock and data analysis. Time-to-event outcomes were analysed using the log-rank test. Hazard ratios (HRs) with 95% confidence intervals (CIs) were estimated using Cox proportional hazards regression. The proportional hazards assumption was assessed by including an interaction term between log(time) and treatment group in the model. Binary outcomes were analysed using Fisher’s exact test, presenting relative risks (RRs) with 95% CIs as effect size. As the 30-day composite endpoint did not show statistical superiority for the HOPE group (hazard ratio [HR] 0.56 (0.32–0.99), log-rank test, *P* = .059), the present 12-month analysis should be regarded as hypothesis-generating rather than confirmatory. The pre-specified efficacy analyses included any patients who were randomly assigned, fulfilled inclusion and exclusion criteria, and received a transplant in the trial (101 patients in the HOPE group and 103 patients in the SCS group). None were lost to follow-up. Baseline characteristics for donors and recipients were well balanced and reported together with the 30-day results.^1^

At 12 months after transplantation, the incidence of the composite efficacy outcome was observed in 32.7% in HOPE vs 46.6% in SCS as depicted in *Figure 1* (HR 0.62; 95% CI 0.40–0.96 *P* = .042). A complementary analysis, adjusted for randomization strata (donor age, donor sex, recipient age, recipient gender, and recipient preoperative use of ventricular assist device), showed consistent results (HR 0.59; 95% CI 0.38–0.93 *P* = .022). The components of the composite endpoint were observed in the following incidences for HOPE and SCS, respectively: death 7.9% vs 13.6% (HR 0.57; 95% CI 0.24–1.36), severe PGD 5.0% vs 20.4% (RR 0.24; 95% CI 0.10–0.62), moderate PGD 5.0% vs 5.8% (RR 0.85; 95% CI 0.27–2.70), PGD right ventricle 4.0% vs 8.7% (RR 0.45; 95% CI 0.14–1.42), ACR >1R 13.9% vs 14.6% (HR 0.94; 95% CI 0.46–1.95), graft failure 2.0% vs 1.0% (HR 2.03; 95% CI 0.18–22.41), CAV 3.0% vs 5.8% (HR 0.48; 95% CI 0.12–1.91). No patient was re-transplanted during the period. Graft failure, defined as the need for temporary circulatory support initiated after postoperative day 1, occurred in two HOPE group patients and one SCS group patient. The incidence of major adverse cardiac transplant events—PGD, rejection, or graft failure all as defined in the primary end point, acute onset of ventricular arrhythmia, cardiac arrest, or multi-organ failure secondary to cardiac allograft failure- in the safety population, consisting of all transplanted patients analysed as treated, was higher in the SCS group 42.4% vs HOPE 25.8% (RR 0.61; 95% CI 0.41–0.90). In the SCS group, 21/103 (20.4%) patients were diagnosed with severe PGD, 9 (43%) died within 12 months. In the HOPE group, 5/101 (5.0%) patients were diagnosed with severe PGD, 2 (40%) died within 12 months.

**Figure 1:**
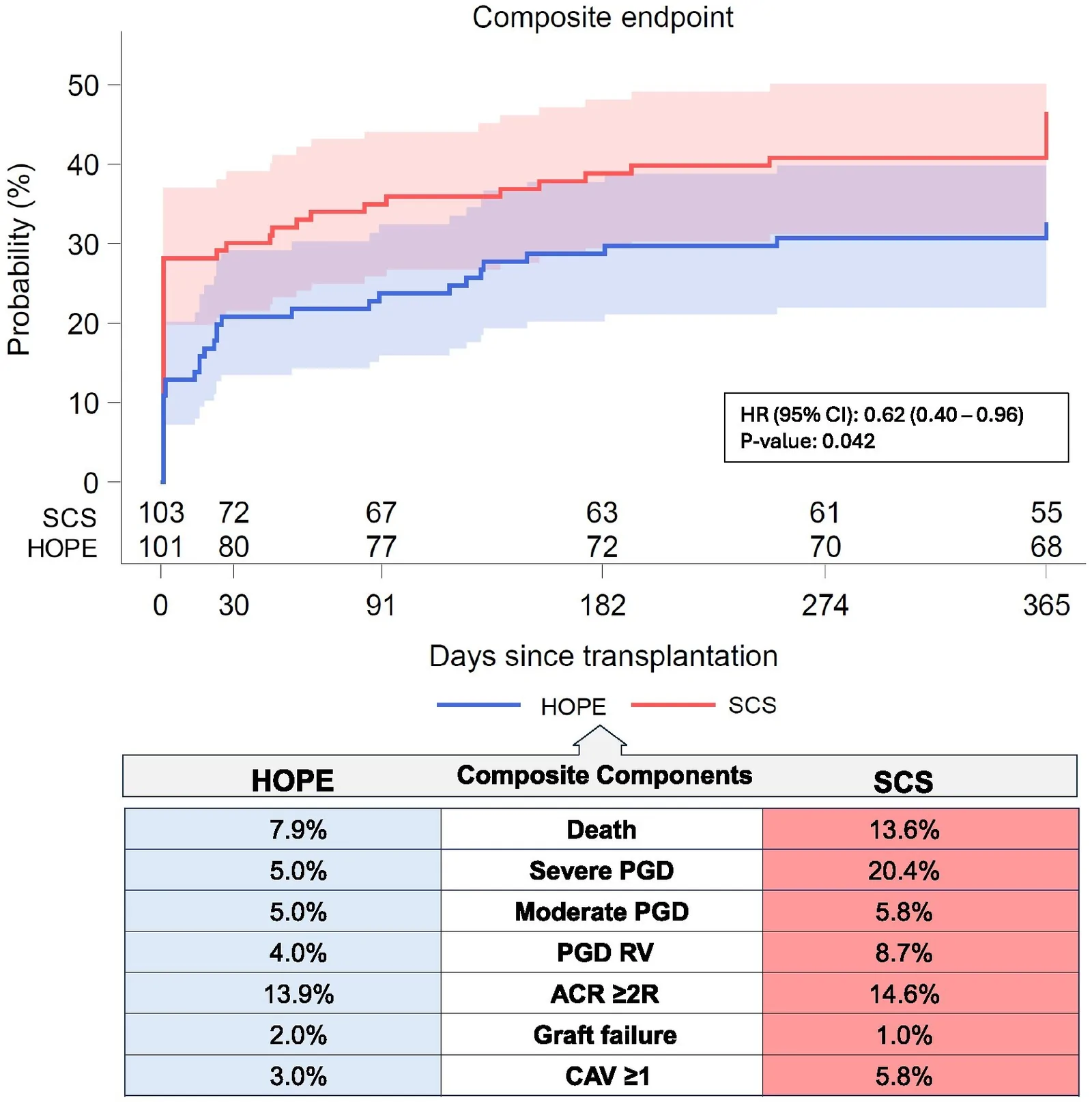
Survival analysis for the composite endpoint during 12 months of follow-up. As specified in the trial protocol, all registered acute cellular rejection events and cardiac allograft vasculopathy events that occurred within the window for the scheduled 12-month visit (11–14 months) were assigned to the 12-month visit in the analysis. *P*-value from log-rank test. HOPE, hypothermic oxygenated perfusion; HR, hazard ratio; SCS, static cold storage; ACR, acute cellular rejection; CAV, cardiac allograft vasculopathy; PGD, primary graft dysfunction; RV, right ventricle

In the SCS group, 200/570 (35.1%) biopsy events in 62 patients (60.2%) indicated any grade acute cellular rejection vs 191/557 (34.3%) biopsy events in 61 patients (60.4%) in the HOPE group. Antibody-mediated rejection was identified in 13 patients in the HOPE group and 13 patients in the SCS group (12.9% vs 12.6%).

This 12-month analysis of the NIHP2019 trial demonstrates that HOPE is associated with improved clinical outcomes compared to static cold storage. The previously reported results at 30 days after transplantation showed a reduction in early patient complications when HOPE was used.^1^ The present findings appear to confirm a reduction in complications throughout the first year after transplantation. The mortality risk associated with severe PGD and the observed reduction with HOPE demonstrate how mitigation of early graft injury through optimal organ preservation can enhance post-transplant outcomes. Moreover, the incidence of major adverse cardiac transplant events within 12 months post-transplant was lower in the HOPE group.

The HOPE donor cohort in the present study reflect the European context, with relatively high age (median 48 years) and often extended graft preservation durations (median 240 min). Both factors are independently associated with inferior post-transplant outcomes, with a notably greater risk when combined.^8^ Recent International Society for Heart and Lung Transplantation recommendations even suggest that hearts from donors ≥45 years should only be used when ischaemic times are expected to be <4 h.^9^ The observed reduction in severe PGD and major adverse cardiac transplant events with HOPE indicates that the technology provides benefit, particularly in challenging donor and recipient scenarios.

Although the ISHLT consensus definition of PGD has faced criticism, the association between PGD and adverse clinical outcomes is well established, particularly for patients diagnosed with severe PGD.^10^ Our findings reinforce this relationship, demonstrating poor 12-month survival in patients with severe PGD. As this association was consistently observed across both study arms, it affirms the reliability of the blinded adjudication process employed. The high 92% survival observed in the HOPE group at 12 months was accompanied by a lower incidence of severe PGD, suggesting an association between improved early graft function and favourable clinical outcome.

HOPE of donor hearts is associated with improved clinical outcomes and reduced postoperative complications at 12 months after transplantation and have potential to improve hospital efficiency and resource allocation. Future research may investigate use in diverse clinical settings, including donation after circulatory death and paediatric transplantation.

## Declarations

### Data Availability

The de-identified study data and data dictionary will be made available to researchers on request. We will provide de-identified individual participant data to fellow researchers. The trial data have been acquired, managed, and archived in compliance with Good Clinical Practice (GCP) and ISO14155:20 standards. Formal data sharing requests are carefully considered, taking into account the guidelines of the study centres and sponsor. Those interested in obtaining data should contact the corresponding author, providing details about the proposed research and the extent of data requirements. To access the data, recipients must enter into a formal data sharing agreement outlining the conditions for release and specifying requirements for data transfer, storage, archiving and publication. Data sharing is approved if the proposed projects demonstrate a sound scientific or patient benefit rationale, subject to endorsement by the independent Data Safety and Management Board. To uphold patient confidentiality and consent, restrictions are imposed by aggregating and anonymizing identifiable patient data. Furthermore, all indirect identifiers that could potentially lead to deductive disclosures will be systematically removed.

### Pre-registered Clinical Trial Number

The trial is registered with ClinicalTrials.gov (NCT03991923)
